# Sharing interim trial results by the Data Safety Monitoring Board with those responsible for the trial’s conduct and progress: a narrative review

**DOI:** 10.1186/s13063-017-1858-y

**Published:** 2017-03-09

**Authors:** Victoria Borg Debono, Lawrence Mbuagbaw, Lehana Thabane

**Affiliations:** Department of Health Research Methods, Evidence, and Impact (HEI), McMaster University, Hamilton, Ontario Canada

**Keywords:** Data Safety Monitoring Board, Data Monitoring Committee, Interim data sharing, Narrative review

## Abstract

**Background:**

Sharing interim data, results or result extrapolations is an important issue that can affect trial integrity. The different ways in which Data Safety Monitoring Boards (DSMBs) share interim results with non-DSMB members and the acceptability of such practices are poorly understood. Our objective was to undertake a narrative review specifically on what kind of interim results, if any, should be shared by the DSMB with non-DSMB members and why.

**Methods:**

We conducted a narrative review using a systematic search strategy of several databases and major health research stakeholders. Literature was included if there was some discussion within the full text about sharing interim trial results with non-DSMB members.

**Results:**

About 79.6% (129/162) of included citations were based on author’s views, 16.7% (27/162) on research guidelines and 3.7% (6/162) on surveys. The largest group of citations, 73/162 (45%), expresses the opinion or argument against sharing interim results with exceptions. Trailing closely, 71/162 (43.8%) of the included citations support the opinion or argument that interim results should not be shared and should remain confidential with the DSMB. Half of the six surveys support sharing in some capacity, while the other three do not. Eleven circumstances were found that potentially warrant interim result sharing by the DSMB; they relate to (1) usual practices by DSMBs, (2) trial completion threatened, (3) patient safety, (4) regulatory approval and (5) other circumstances. Dominant risks for sharing under these conditions are associated with introducing trial bias.

**Discussion/conclusion:**

There was no majority view in the literature. However, the largest group of citations included express the idea that interim results should remain confidential with the DSMB but also acknowledge circumstances when they could be shared with non-DSMB members. Limitations of this review are that (1) the included literature predominately provides personal perspectives, not evidence, and (2) surveys found globally focus on trial monitoring practices lacking detailed information on what specifically to share, with whom and why. More research is needed with the use of a detailed survey of the clinical trial community focused on DSMB sharing interim results, to better understand and guide DSMB interim result sharing practices.

**Electronic supplementary material:**

The online version of this article (doi:10.1186/s13063-017-1858-y) contains supplementary material, which is available to authorized users.

## Background

The Data Safety Monitoring Board (DSMB) or the Data Monitoring Committee (DMC) is responsible for the stewardship of a trial. This group can help oversee the safety of patients in the trial by looking at unmasked safety or efficacy data to make recommendations to the Steering Committee (SC). They can also oversee, in sequential designs, if the trial results have reached the predefined amount of information needed to finish the trial. Importantly, they also protect the trial from the bias that could be introduced during the trial conduct [1, 2].

The case that triggered us to assess further this issue of sharing interim trial information was described by Anand et al. [3] when the funding sponsor asked the SC and DSMB of a cardiovascular trial to provide them with interim adaptive conditional power before approving the request for additional funding requested by the investigators. Adaptive conditional power “is the probability that the trial will reach statistical significance if continued to completion if the difference specified in the trial protocol is true, given the outcome events that have already been observed, and the time remaining to observe additional events among patients who are currently event free” [3, 4]. It is a result extrapolation based on interim relative efficacy results [2]. The DSMB refused to give this information because they considered that sharing adaptive conditional power would unmask the trial’s interim results.

When we consider a review done by Grant et al. back in 2005 [5] that globally looks at many issues related to data monitoring and interim analysis, we see that there seems to be an accord that interim results and DSMB deliberations remain confidential. However, the review are also mentions instances where interim results may be shared with the independent unmasked statisticians or other individuals such as the chair of the SC if the DSMB deems it best to do so because of a safety issue. See the Discussion section for a further discussion of this review. Still, the review lacks certain details such as the specifics of what kind of interim results are shared and with whom. Sharing of interim trial data, results or result extrapolations by the DSMB with individuals who are non-DSMB members, who are responsible for the conduct of the trial, can negatively affect trials [6]. One of the major concerns related to sharing interim trial results [1] is the potential for non-DSMB members to consciously or subconsciously introduce bias that will affect the final trial’s results [1, 7]. This is an especially important issue for phase III trials, which are usually designed and used to find definitive evidence on efficacy and safety endpoints to inform practice or for regulatory drug approvals [1, 2]. This case [2] and the review [5] brought to mind the following questions: Is it possible that there are other circumstances where the DSMB is justified in sharing interim data, results or extrapolations with non-DSMB members? If so, what information would be shared in such circumstances and with whom?

The overall objective of this review and commentary is to (1) provide a summative narrative review of the views and opinions on the issue of the DSMBs sharing interim results during the conduct of a clinical trial, particularly phase III trials, with the Principal Investigators (PIs), the sponsor, the SC, other parties responsible for the conduct of the trial or any other non-DSMB member(s); and (2) discuss what interim data, results or result extrapolations the DSMB should share, if anything at all, with whom and under what circumstances. The information required to inform this narrative review was gathered from a systematic literature search. For simplicity, the remainder of this review will refer to any assortment of interim data, interim results or interim result extrapolations as interim results. Throughout the rest of the review we will also refer to PIs, the sponsor, the SC, investigators, site managers, independent unmasked statisticians, the funder(s), or patients enrolled in the trial or any other party responsible for the conduct or completion of the trial as non-DSMB members; we will be more specific when needed.

## Methods

A narrative review was considered the most appropriate method to use because it allowed us to explore the literature and inductively evaluate qualitative and quantitative information. We anticipated that most of the literature we would find would be opinions, policies or guidelines, and a narrative review would require an inductive approach for theme analysis and categorisation. To find literature discussing the issue of DSMBs sharing interim results, a broad and comprehensive systematic search of the literature was done in December 2015 within the databases of PubMed (includes all MEDLINE citations), Web of Science, EMBASE and CINAHL from the inception of all four databases using a detailed search strategy for each of them, as outlined in Additional file 1. Key phrases related to ‘Data Safety Monitoring Boards’ were utilised in each of the four databases as well as a filter for articles in the English language.

The title and abstract for each citation that came up within the search of each of the four databases were reviewed. Citations were eligible and included for full-text review if the title or abstract associated with a particular citation met the following inclusion criteria: [(1) related to DSMB issues OR (2) related to the management, operation, conduct, use, experience, or discussion of DSMBs] AND (3) the article associated with the citation was published in English. Subsequently, citations from the full-text review were eligible and included for full-text information extraction if there was some focused discussion or statement, within the full-text, about sharing interim trial results with parties outside of the DSMB. Reference lists from included articles were searched for other unique articles discussing the issue of DSMBs sharing interim trial results using the same inclusion criteria. Additionally, two major textbooks that solely focused on the operation and management of DSMBs were also reviewed and consulted [1, 2]. These were found upon discussion with a professor who is a health methodologist with expertise in clinical trials.

We also searched regulatory, governmental and guideline groups from the USA, Canada, UK, European Union and Australia, and two international groups, the International Conference on Harmonisation of Technical Requirements for Registration of Pharmaceuticals for Human Use (ICH) [8] and the World Health Organisation (WHO) [9]. For a list and details of the organisations that were searched directly for relevant literature or information from their respective websites, see Additional file 1. Two strategies were used in combination to find relevant literature on the issue of interim result sharing by the DSMB within major governmental/regulatory/funding bodies and guideline groups (Additional file 1). Documents and webpages on an organisation’s website that had information about clinical trial research and DSMBs, as indicated from a webpage’s or document’s title or within the full text, were eligible and included for full-text information extraction. A full-text review was done immediately because most webpages or documents found on these organisation websites are not structured like most journal articles with an abstract. Documents and pages from the full-text review were eligible and included for full-text information extraction if there was some focused discussion or statement within the full text about sharing interim trial results with parties outside of the DSMB. Please note that within the Results and Discussion sections, not all the citations included from our systematic search of the literature for this review are cited in this paper. A full citation list of articles found to support this review in its entirety is given in Additional file 2. All screening and full-text extraction was done independently by one of the authors (VBD) for this narrative review and then checked by co-reviewers (LM and LT). Information extracted from the included full text pertained to the sharing of interim trial results with parties outside of the DSMB. Any disputes or concerns were resolved by consulting co-reviewers (LM and LT). We worked inductively and thus retrospectively with the included literature to perform a categorisation and thematic literature analysis. Analyst triangulation was used among co-reviewers (LM and LT) with VBD to ensure that categorisation and thematic analysis of literature were sound. No changes were made to the review process that was initially planned. Our review is principled in pragmatism review because we were not initially sure what categories or themes would emerge from the literature. Please see the RAMESES checklist [10] attached as Additional file 3.

## Results

**Fig. 1:**
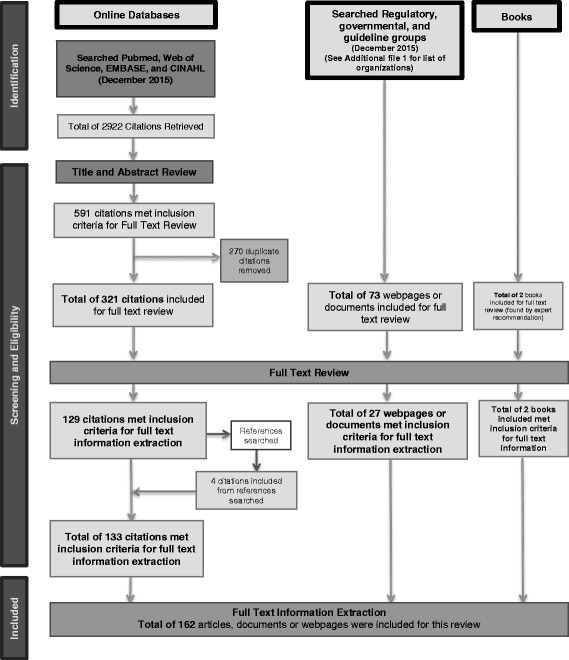
Flow diagram of the literature inclusion process

A total of 162 articles, documents, policies, guidelines books or webpages were included for this review. See Fig. 1 for a flow diagram showing the inclusion process and the number of articles included.

There are mixed views and opinions on the issue of the DSMBs sharing interim results during the conduct of a clinical trial with non-DSMB members. Out of the 162 included articles, 129 (79.6%) were based on author’s views, 27 (16.7%) on DSMB or trial research guidelines and 6 (3.7%) on surveys of trialists. The literature falls into three categories of opinions: Category 1 - literature that expresses the opinion or argument *against* sharing interim results, stating that they should remain confidential with the DSMB (71/162, 43.8%); Category 2 - literature that expresses the opinion or argument *against* sharing interim results with *exceptions* (73/162, 45.0%); and Category 3 - literature that expresses the opinion or argument *in favour* of sharing interim results by the DSMB with a non-DSMB group (18/162, 16.8%). No one group held the outright majority. However, the largest two literature groups that were very close percentage wise were Category 1 (43.8%) and Category 2 (45.0%).

Six surveys were also found out of the 162 included articles that looked globally at trial monitoring practice as described in detail in Table 1. These surveys did not specifically focus on the issue of DSMBs sharing interim results during the conduct of a clinical trial with non-DSMB members. However, all of them asked at least one question that was related to the issue and surveyed those who were somehow involved in trials. Two of the six surveys report results that are quantitatively unclear, as their results are qualitatively described. The remaining four of the six surveys report results quantitatively. The target populations for these surveys varied, ranging from directors of the statistical centres from 12 cancer cooperative groups sponsored by the National Cancer Institute (NCI) in the USA from 1993 [11], trialists of past and ongoing trials [5, 12], PIs and biostatisticians on DSMBs and Institutional Review Board (IRB) community representatives [13], major funders of trials, regulatory agencies and other relevant organisations related to trial research [5]. Methods to obtain the sample also varied. Sampling frames included statistical centres from 12 cancer cooperative groups sponsored by the NCI in the US in 1993 [11], the US National Institutes of Health (NIH) [12], ClinicalTrials.gov, a MEDLINE search of articles pertaining to randomised controlled trials (RCTs) from Biometrics or Statistics in Medicine, the Office of Human Research Protection website [13], a database of Health Technology Assessment (HTA) programme and Medical Research Council (MRC) trials [5] and a list of 25 handpicked organisations that are major funders of trials, regulatory agencies and other relevant organisations related to trial research [5]. Response rates ranged from 40% to 100% [5, 11–13]. The number of responses to these surveys varied from 12 to 309 [5, 11–13]. Respondents were not chosen at random in two cases [11, 12]. The other four surveys had some method to select respondents at random [5, 13]. Survey collection methods included telephone interviews [5], email surveys [5] and mail/paper surveys [5, 11–13].

**Table 1:** Surveys looking globally at trial monitoring practices

Year of survey	Reference	Sampling frame	Number of people to whom survey was sent (*n*) and survey response rate	Number of people (*n*) who answered the question related to interim data or results sharing	Results	Interpretation of their results
1993	[12]	Trialists from the NIH for the USA	*n* = 12 Response rate: 100%	*n* = 12	Quantitative results are unclear Only qualitatively reported as: “DMC reports are confidential, with access to DMC members and selected institute staff.”	Respondents from the NIH support that DSMB reports are to be confidential and privy only to DSMB members with access also granted to selected NIH US staff.
1993	[11]	Directors of the statistical centres from 12 cancer cooperative groups sponsored by the NCI in the USA	*n* = 13 Response rate: 100%	First question about reporting interim results by treatment regimen: *n* = 9 Second question related to non-DSMB member access to interim data reports: *n* = 10	First question: 0% of the respondents (0) indicated that NCI groups provided unmasked outcome reports to the participants. Second question: 70% of the respondents (7) indicate interim data reports are not accessed by non-DSMB members	The majority of respondents indicate interim data reports are not accessed by non-DSMB members.
2000	[5]	Trialists from completed trials	*n* = 45 Response rate: 62%	Unclear	Quantitative results are unclear Only qualitatively reported as: “Views on sharing the interim information with other DMCs were consistent; the investigators were not enthusiastic about the DMC consulting others”	Based on the qualitative reporting it appears that investigators are not supportive of DSMB consulting others outside of the DSMB.
2002	[5]	Trialists from ongoing trials	*n* = 40 Response rate: 80%	*n* = 20	50% of respondents (10) agree with the DSMB sharing interim data or results, if it is necessary, with non-DSMB members • Many of these 10 respondents said it should be done if there was a safety concern • 1 respondent from this group felt it was acceptable to share safety but not efficacy data • 2 respondents from this group felt that the SC should make the decision if the DSMB were to share data or results on an individual basis 30% of respondents (6) have no provision for the DSMB sharing data or results with non-DSMB members • 1 respondent from this group indicated that the need to share should be dealt with by the SC on an ad hoc basis 20% of respondents (4) disagree with the DSMB interim data or results sharing with non-DSMB members • 1 respondent from this group indicated that the DSMB can seek external advice but not share interim data or results	Variation and disagreement in the responses about whether the DSMB should share interim data or results with non-DSMB members. The largest group of respondents (50%) agree with DSMBs sharing interim data or results with non-DSMB members when it is necessary, particularly for safety.
2001/2002	[5]	Review of DSMB policies of major funders of trials, regulatory agencies and other relevant organisations related to trial research	*n* = 25 Response rate:100%	*n* = 17	All the respondents indicated that some non-DSMB members had access to interim data or results. Who had access to interim data or results was as follows: 41% of respondents (7) indicated that everyone has access to interim data or results except the participants 35% of respondents (6) indicated that key institute staff has access to interim data or results • 1 respondent from this group said there was also provision for interim data to be seen occasionally and confidentially by the DSMB of another trial 18% of respondents (3) indicated that only the trial statistician and the DSMB had access to interim data or results 6% of respondents (1) allow data centre personnel to access interim data or results	All respondents from major agencies that are involved in trials indicate that interim data or results are shared with certain non-DSMB members. The largest minority of respondents (41%) indicate interim data/results are shared with everyone except the ﻿participants.
2011	[13]	PIs and biostatisticians on DSMBs and IRB community representatives	Total *n* = 309 Response rate: 31% from PIs 51% for biostatisticians 40% from IRB community members	*n* = 246 PIs = 152 Biostatisticians = 40 IRB community members = 54	• 100% of Biostatisticians (40) indicate that sponsor should be masked to interim data or results • 78.3% of PIs (119) indicate that the sponsor should be masked to interim data or results • 63.0% of IRB community members (34) indicate that the sponsor should be masked to interim data or results	The majority of PIs and biostatisticians on DSMBs and IRB community representatives believe that the sponsor should be masked to interim data or results. Details about sharing with other non-DSMB members were not discussed

*IRB* Institutional Review Board, *NCI* National Cancer Institute, *NIH* National Institutes of Health for the USA, *SC* Steering Committee, *PI* Principal Investigator, *UK* United Kingdom

Three of the six surveys [5, 11, 13] (one qualitatively and two quantitatively reported) support the view against sharing interim results, stating that they should remain confidential with the DSMB (Category 1 view). For one of the two surveys reported quantitatively [11], all respondents (*n* = 9) indicated that NCI groups, at the time the survey was administered, did not provide unmasked outcome reports to the participants. For a second question in the same survey asking about which non-DSMB members had access to interim data reports [11], it was reported that 70% of the respondents (*n* = 10) indicated that non-DSMB members do not access interim data reports. For the remaining three respondents who answered this question, who alternatively stated that non-DSMB members do have access to interim data reports, there is no mention as to who specifically gets this information and why. For the second survey [13], as can be seen in Table 1, the majority of respondents, all of whom were biostatisticians, PIs or IRB community members, indicate that the sponsor should be masked to interim data or results (percentages can be viewed in Table 1). Masking of other non-DSMB members besides the sponsor was not mentioned. For the minority of respondents who said “No” to this question asking if the sponsor should be masked to interim data or results (0% biostatisticians, 21.7% PIs and 37.0% IRB community members), there was no mention as to why this information should be shared with the sponsor. For the one survey that qualitatively reported their results indicating “Views on sharing the interim information with other DMCs were consistent; the investigators were not enthusiastic about the DMC consulting others” [5], there was no mention of whether there were respondents with another view.

One survey [5] (quantitatively reported) supported the Category 2 view: *against* sharing interim results but with *exceptions*. The largest group of respondents from this survey, for the question related to interim data or results sharing (50%, 10/20), agreed with DSMBs sharing interim results with non-DSMB members if it was necessary, particularly for concerns related to participant safety [5]. Specifically with whom this information would be shared was not clear. For the remaining respondents to this question, 30% (6/20) of respondents had no provision for, or idea about, the DSMB sharing interim data or results with non-DSMB members, and 20% (4/20) of respondents disagreed with the DSMB interim data or results sharing with non-DSMB members.

Two surveys [5, 12] (one qualitatively and one quantitatively reported) supported the view *in favour* of sharing interim results by the DSMB with a non-DSMB group (Category 3 view). For the survey that was reported quantitatively [5], all of the respondents to the question related to interim data or results sharing (*n* = 17) indicated that someone outside of the DSMB had access to interim data or results during their trial. With whom interim results were shared was indicated as follows: 41% of respondents indicated everyone except the participants, 35% of respondents indicated key institute staff (one respondent from this group said there was also provision for interim data to be seen occasionally and confidentially by the DSMB of another trial), 18% of respondents indicated only the trial statistician and the DSMB and 6% stated that the data centre personnel had access to interim data or results. Why information was shared with these non-DSMB members was not discussed. For the other survey [12] that reported results qualitatively, it was indicated that respondents (*n* = 12) from the US NIH support that DSMB reports are to be confidential and privy only to DSMB members. However, access is also granted to selected US NIH staff, these being non-DSMB members. Why information was shared with selected US NIH staff was not discussed.

For the other 158 documents, which were not describing surveys, we assessed for a time trend to see where the views and policies lie for the last ten years, back to 2006, in regard to the three categories we identified. For the literature in Category 1, dating back to 1981, 54% (37/68) of the literature comes from the past ten years alone. For Category 2, the literature dates back to 1998. About 24% (4/17) of the literature comes from the last decade. For the literature in Category 3, dating back to 1991, 52% (38/73) of the literature comes from the past ten years. The most recent literature, count and percentage wise, has predominately supported the DSMB not sharing or not sharing but with some exceptions. We also found that regulation, policy or guideline documents predominately support Category 3 (55%, i.e. 15/27 of the 27 regulation, policy or guideline documents included in our review).

In regard to our second objective, there is a subset of the literature within Category 2 or 3 literature that discusses what interim results the DSMB should share, with whom and the circumstance (why). Eleven circumstances that may warrant the DSMB sharing interim results with non-DSMB members are explained in Table 2, generally categorised under four themes: (1) current usual practice by DSMBs, (2) trial completion is threatened, (3) concern about patient safety and (4) regulatory approval). There is also a category for other special circumstances that includes three unique situations for DSMB sharing of interim results that did not fit into a theme. Six of these eleven circumstances are supported in the literature with real-life examples. What is shared by the DSMB with non-DSMB members varies depending on the particular circumstance. For many of the cases where sharing may be warranted, a risk or counter argument to sharing is indicated where applicable. Most of the risks with sharing in these circumstances are predominately associated with introducing bias in the trial that will affect the final trial results. It is indicated in the literature that there is always the potential that sharing interim results with non-DSMB members may do harm to a trial by disturbing equipoise [14, 15], as people may make inaccurate impressions about what is happening between treatment groups [3, 16–18]. It is explained that when equipoise is disturbed with knowledge of interim results by those operating and managing the trial and those participating in the trial, there may be actions people can take, either consciously or subconsciously [19], that can bias the trial’s results [1, 16, 19]. The introduction of bias can reduce the credibility and integrity [16, 20, 21] of the trial, rendering the results questionable [16, 20, 22].

**Table 2:** Circumstances where interim result sharing may be warranted by the DSMB

Circumstance	With whom would the DSMB share?	What to share?	Risk or counter argument	Reference
Theme 1) Current usual practice by DSMBs				
Circumstance 1: When the DSMB recommends early termination and the recommendation needs to be evaluated by the SC and sponsor	Specified representative(s) of their trial’s SC and sponsor	Unmasked interim results	Risk: If the trial were to continue despite the recommendation to terminate, those few individuals privy to the interim data should not be a part of making future trial decisions. This will protect the trial’s integrity from potential biasing of results	[6]
Circumstance 2: When the DSMB has concerns about the interim data or results given to them by the unblinded independent statistician or DAC for their interim review	Trial’s independent statistician or DAC	Anything needed	None made	[16, 20, 27–38]
Theme 2) Trial completion is threatened				
Circumstance 3: When the trial may have to stop early because of poor accrual due to special circumstances, and it may be possible to improve accrual by sharing interim data or results, when all other efforts to improve accrual are exhausted	The public	Some type of unmasked interim result that will encourage accrual	Risk: Risk of biasing trial results even when special conditions are met as indicated by Stephens et al. [39]. Sharing interim results should be a judgement call that weights the benefits of sharing against the potential risk of biasing trial results	[16, 30, 39–43] [39]*
Circumstance 4: When there is a need to restore equipoise when one of two related trials finishes first and threatens the completion of the unfinished trial	The public	Sharing unmasked but limited comparative interim results that will help restore equipose	Counter argument: The unfinished trial(s) might not need to share interim information if it will contribute important information beyond what was reported by a similar trial that finished earlier. This sentiment should be expressed to all stakeholders to help restore confidence in trial completion	[1, 16, 44] [1]*
Theme 3) Concern about patient safety				
Circumstance 5: When an uncertain severe safety issue appears at interim in a trial and there is another similar trial still underway	The DSMB of the similar trial	Safety: Unmasked interim safety result	Risk: Sharing may erode the independence of each trial in regards to the independent confirmation of results	[1, 5, 6, 17, 23, 44–52] [1]*
Circumstance 6: When the DSMB assesses the risk of there being a serious adverse event at interim for enrolled patients in a particular treatment group, but continuing the trial may still be desirable because getting a definitive result on a patient primary endpoint is important to the public and medical community	Trial patients	Safety: Unmasked interim safety results	Risk: Unmasking of interim safety results with the trial patients may risk biasing the trial results, but in some cases it is ethically imperative to let the patients know of the severe safety risks to allow them to decide whether they want to continue in the trial and before allowing the trial itself to continue	[1, 14, 18, 53, 54] [1]*
Theme 4) Regulatory approval				
Circumstance 7: When the regulator is currently assessing licensing approval for a new drug/treatment submitted with results from a completed trial and there is still a similar trial underway that will provide important new substantial information regarding results	The regulators	Relevant unmasked interim results that will help with assessing the status that should be given for a licensing application	Risk: Interim review of the second ongoing trial could jeopardise its own integrity and introduce bias, as the public could prognosticate the results of that trial based on the regulator’s subsequent decision to either approve or delay a manufacturer’s licensing application	[1, 20] [1]*
Circumstance 8: When a regulatory wants to assess a drug for conditional or accelerated/expedited approval for a manufacturer to be able to market a drug early	The regulators	Unmasking interim results	Risk: Bias could also be introduced to the trial with knowledge of regulatory decisions made based on interim results and known threshold criteria for approval, even if exact interim endpoints are not shared publically	[1, 5, 16, 28, 55–61] [57, 60]*
Other special circumstances				
Circumstance 9: When adaptive confirmatory trials base interim trial adaptive changes on the trial’s interim results	Authorised qualified persons at the sponsor (1 or 2 people) who are not participating in the trial but can assist with trial adaptations	Whatever is agreed upon a priori	Risk: Unmasking of interim data or results can introduce bias and risk trial integrity	[30, 62–68]
Circumstance 10: When patients outside of the trial are facing important treatment decisions and may benefit from some interim results from non-inferiority or superiority trials with a long follow-up	The public and patients and physicians facing important treatment decisions	Relevant unmasked interim results that will help with treatment decision	Risk: Knowledge of an interim endpoint result could influence a clinical decision to have a new treatment before safety of that treatment is determined more definitively in ongoing trial	[11, 69, 70]
Circumstance 11: When sponsors, investigators or regulators are planning for future studies, new products or allocating resources for future use	Sponsors, investigators or regulators	Unmasked yet non-comparative interim information. This could be: • Control group event rates OR • Control group adverse event rates OR • Pooled event rate	Risk: Bias can be introduced to the unfinished trial if new plans are to be published and can be interpreted by a wider audience. Planning errors could result from using uncertain interim results	[1, 6, 20, 71–74]

*DAC* Data Analysis Centre, *DSMB* Data Safety Monitoring Board, *SC* Steering Committee

Circumstances 3–8 have a real-life example and an asterisk (*) next to the associated reference(s) with the example

## Discussion

### What are the findings from the review?

We found three main views on the DSMB sharing interim results with non-DSMB members. These views were (1) *against* sharing (Category 1), (2) *against* sharing interim results with *exceptions* (Category 2) and (3) *in favour* of sharing interim results (Category 3). The literature predominately supported Category 1 and Category 2 in similar proportions. We found that the literature in Category 2 and Category 3 presented 11 cogent reasons for sharing interim results by the DSMB with various non-DSMB members in certain circumstances (Table 2). The three surveys [5, 12] that support the Category 2 and Category 3 views do not specifically indicate what should or should not be shared in circumstances that may warrant sharing interim results. However, one of the surveys [5] that support Category 2 indicates that the DSMB having a safety concern is a circumstance that justifies the DSMB sharing interim results with non-DSMB members. In these 11 circumstances, what is shared and with whom depends on the circumstance. Six of the 11 circumstances (see Table 2) have real-life examples (anecdotal evidence) where interim result sharing by the DSMB with a non-DSMB member helped the trial and the patients who were enrolled. However, for most of these 11 circumstances, there are risks acknowledged—mainly in regard to introducing bias that may affect the trial’s final results. Based on these 11 circumstances that may occur, there is possible legitimacy in the notion that what may or may not be shared and with whom at interim should be a judgement call made by a trial’s DSMB, where the DSMB as a group balances the apparent risks and benefits in those circumstances that arise regarding the need to protect both the safety of the patients enrolled and the trial’s integrity. Sometimes the DSMB may find that the benefits of sharing seem to outweigh the risks, as was illustrated by some real-life examples that supported 6 of the 11 circumstances. The DSMB may also find that the risks of sharing are not worth the benefits that could result. The opinion of the DSMB sharing interim results is also supported in part by both Chalmers et al. [23] and Shah et al. [24]. Chalmers et al. [23] comment on the need to share interim results when the occasion is appropriate and to identify and plan for such situations a priori when possible.

### How do these findings compare with those of similar works?

While our review is unique in that it solely focuses on the issues of interim result sharing by the DSMB with non-DSMB members, another review, done earlier in 2005 by Grant et al. [5] under the auspices of the National Health Service (NHS) in the UK, looks globally at many issues related to data monitoring and interim analysis. One of 23 questions they ask addresses in part the issue with DSMB confidentiality of interim data. They found within their literature review under *Question 8: Should the DMC deliberations be open or closed (confidential or secret as opposed to publicly available)?* that “There is near unanimity that the interim data and the deliberations of the DMC should be absolutely confidential” [5], which supports what we found in our review with Category 1 literature which held a large percentage of the literature reviewed (43.8%). However, a sub-question to their Question 8 asked: *Who outside the committee should see the interim analysis and how is this changed by whether the analyses were blinded or unblinded?* They indicate that some have suggested that an independent unmasked statistician should see the interim analysis [5], which is what we found for Circumstance 2 described in our Table 2. They also indicate that the DSMBs may allow certain individuals such as the chair of the SC to become unmasked to certain interim results, especially if this is deemed by the DSMB of the trial to serve patient/participant safety best [5]. For Circumstances 5 and 6 in Table 2, we also find that a severe safety issue in the trial is a potential driving factor to share unmasked interim safety results with non-DSMB members. Thus, their review [5] also supports what we found in Category 2 literature: even though interim results should remain confidential with the DSMB, there are circumstances the DSMB must consider that could warrant interim sharing outside of the DSMB. Our review goes into detail about the circumstances that may warrant sharing (see Table 2), and we do our best to summarise all the views on this issue. Our intent was also to discuss what to share and with whom in those circumstances and, as described above, we found that it greatly depends on the circumstance in which the DSMB finds itself, in regard to the trial.

### What are the key limitations?

One limiting factor of this review is that the majority of the included literature was based on personal perspectives (79.6%). Though these perspectives brought up important points, these views represent a small fraction of all the professionals who are involved in trials and are not based on evidence. Many of these views contributed to describing the 11 circumstances found in Table 2, where only 6 out of the 11 circumstances were supported by anecdotal evidence. The other 7 circumstances were based on the author’s experience or view that would warrant sharing by the DSMB if the DSMB deemed it necessary and safe to do so.

Another limitation is that the six surveys do not fully help us with our objective to understand, with empirical evidence, what interim results, if any, the DSMB should share, with whom and under what circumstances. They globally focus on trial monitoring practices and not in depth or specifically on the issue of DSMB sharing interim results. At most, one or two questions within each survey ask a question related to the DSMB sharing interim results with other non-DSMB groups, but the questions are not asked in a consistent way for all the surveys. For instance, for three of these surveys, the questions related to interim result sharing asks respondents about their current practices regarding who has access to unmasked outcome reports [5, 11, 12] at their research institutions. For another survey, the question very specifically asks respondents if the sponsor should be masked to interim results [13]. For two of the surveys [5], the question related to interim result sharing asks respondents if they think unmasked interim results should be shared with non-DSMB members. So, although we can understand overall where support lies from each survey in regard to sharing interim results, and in some surveys we have a bit of information about with whom interim results may be shared, it is still extremely unclear what specific kind of interim result should be shared, with whom that result should be shared and for what reason or circumstance. There is not one survey that consistently asks the same group of respondents who are trialists, what interim results specifically should be shared, with a direct follow-up question about who outside of the DSMB should share that specific interim result and for what reason (why). This is a complex topic. It is challenging for any survey soliciting responses about global trial monitoring practices to use one or two questions to sufficiently address and provide enough information clarifying what interim results the DSMB should share, if anything at all, with whom, and why. Multiple questions or an entire survey dedicated to the topic of DSMB interim result sharing is needed.

### Implications of the findings

Our findings inform trialists and those who enact trial policies and guidelines that there are mixed views on the DSMB sharing interim results with non-DSMB members. We could argue that out of the three categories, the literature in Categories 1 and 2 dominates in that the DSMB should not share interim results with non-DSMB members, but the literature in Category 2 suggests that there may exceptions. The exceptions include 11 possible circumstances as described in Table 2. However, the findings from this review need to be substantiated with more research. The empirical evidence found within three of the six surveys [5, 13] suggests there is support for sharing interim results with certain non-DSMB members, but the details on what specifically should be shared and for what reason are unclear. Based on the limitations described in the previous section, more empirical evidence is needed to clarify specifically what interim results should or should not be shared by the DSMB with non-DSMB members, with whom and for what reason or circumstance, to better inform monitoring practices, policies and guidelines that protect the safety of the participants enrolled and trial validity.

For the situation described earlier by Anand et al. [3], we also question: How useful is it to share unmasking yet non-comparative interim results (e.g. control group event rates without knowledge of the pooled events rates)? Also, how useful is it to share results that appear masking of comparative results (e.g. adaptive conditional power or aggregate/pooled results by treatment group)? It is thought that knowledge of aggregate/pooled results can lead to concerns about making assumptions about interim results [25, 26] that are not necessarily true, which could lead to introducing bias in the trial. Knowledge of such potentially unmasking information, such as the adaptive conditional power, could jeopardise the integrity of the trial, as it does indicate the probability of the trial showing a favourable significant result [2]. In the case described by Anand et al. [3], the request for the adaptive conditional power by the trial sponsor was denied by the trial’s DSMB, and the decision to not share was additionally supported by the trial’s SC, PI and others outside of the trial who were consulted. There was also mention of the DSMB sharing an ‘unconditional’ conditional power with the sponsor. This ‘unconditional’ conditional power calculation [3] was shared with the sponsor because it is thought to mask the efficacy results if given out at interim, but also provide reassurance to the sponsor that the trial will have the power to answer the primary hypothesis initially set out at the design stage of the trial, when the trial is completed [3]. Is providing the ‘unconditional’ conditional power a helpful alternative to sharing aggregate/pooled results? Should a result extrapolation such as aggregate/pooled results or adaptive conditional power be shared? How is such information interpreted? The issue of sharing aggregate/pooled interim results needs further investigation. More clarity is also required on the specifics of sharing aggregate/pooled interim results, particularly interim results that are thought to be masking, such as the combined event rate, and result extrapolations, such as adaptive conditional power, that have been requested in the past [3].

## Conclusions

Interim result sharing is an important issue because it affects the validity of the results from confirmatory trials on which we base regulatory and practise decisions, impacting the health and lives of many. From this review, two categories of the literature dominate (Category 1 and Category 2), but not in majority as distinct groups. Category 1 is against sharing interim results, stating that they should remain confidential with the DSMB, and Category 2 shares the same sentiment as Category 1 but additionally acknowledges exceptions, that there are circumstances that may warrant the DSMB to share interim results with certain non-DSMB groups/members. What is shared with these non-DSMB members depends on what the situation calls for and should be assessed by the DSMB using their expertise to balance risk(s) with the potential benefit(s) regarding participant safety and trial validity and integrity. Because of the limitations of the evidence found, collecting more empirical evidence through a survey of the general clinical trials community focused on the issue of DSMB sharing interim results (what, if any, interim result(s) to share, with whom and under what circumstance) is needed to better understand and guide DSMB interim information sharing practices.

## Additional files

Additional file 1: Search strategies for literature. (DOCX 33 kb)
Additional file 2: Citations of included articles for review. (DOCX 33 kb)
Additional file 3: RAMESES checklist. (DOCX 18 kb)

## Abbreviations

CRO: Contract Research Organisation
DAC: Data Analysis Centre
DMC: Data Monitoring Committee
DSMB: Data Safety Monitoring Board
FDA: Food and Drug Administration
IRB: Institutional Review Board
ISC: Independent Statistical Centre
NCI: National Cancer Institute
NHS: National Health Service
NIH: National Institutes of Health
PI: Principal Investigator
RCT: Randomised controlled trial
SC: Steering Committee
SDAC: Statistical Data Analysis Centre
SOP: Standard Operating Procedure
UK: United Kingdom
