# Supplementary material for: Sharing interim trial results by the Data Safety Monitoring Board with those responsible for the trial’s conduct and progress: a narrative review
Source: Trials. 2017 Mar 9;18:120. doi: 10.1186/s13063-017-1858-y (PMC5345177; doi:10.1186/s13063-017-1858-y)
Supplement: Additional file 1: — Search strategies for literature. (DOCX 33 kb) [file 13063_2017_1858_MOESM1_ESM.docx]

**Additional file 1: Search Strategies for Literature**

**Search Strategy for PubMed (December 2015)**

The *PubMed* (includes all *MEDLINE* citations) database was searched for all articles pertaining to the discussion of Data Safety Monitoring Boards from 1946, with some older material, to December 2015.

896 citations were found using the search strategy below.

The following was the search strategy used:

| **Search Number** | **Number of**  **Citations Found** | **Search Query** |
| --- | --- | --- |
| #2 | 896 | Search ("english"[Language]) AND #1 |
| #1 | 934 | Search "data monitoring committees"[Title/Abstract] OR "data monitoring committee"[Title/Abstract] OR "data monitoring boards"[Title/Abstract] OR "data monitoring board"[Title/Abstract] OR "data safety monitoring boards"[Title/Abstract] OR "data safety monitoring board"[Title/Abstract] OR "data safety monitoring committee"[Title/Abstract] OR "data safety monitoring committees"[Title/Abstract] OR "data safety and monitoring boards"[Title/Abstract] OR "data safety and monitoring board"[Title/Abstract] OR "data and safety monitoring board"[Title/Abstract] OR "independent data monitoring boards"[Title/Abstract] OR "independent data monitoring board"[Title/Abstract] OR "independent data monitoring committees"[Title/Abstract] OR "independent data monitoring committee"[Title/Abstract] OR "independent data safety monitoring committees"[Title/Abstract] OR "independent data safety monitoring committee"[Title/Abstract] OR Clinical Trials Data Monitoring Committees [Mesh Terms] OR data and safety monitoring boards[MeSH Terms] |

**Search Strategy for Web of Science (December 2015)**

The *Web of Science* database was searched for all articles pertaining to discussion of Data Safety Monitoring Boards from inception of the database to December 2015, Timespan: All years. Indexes: using the Web of Science Core Collection: Citation Indexes, specifically the Science Citation Index Expanded (SCI-EXPANDED) --1976-present.

627 citations were found using the search strategy below.

The following was the search strategy used:

| **Search Number** | **Number of**  **Citations Found** | **Search Query** |
| --- | --- | --- |
| #3 | 627 | #2 OR #1  Indexes=SCI-EXPANDED Timespan=All years |
| #2 | 627 | (TS=("data monitoring committees" OR "data monitoring committee" OR "data monitoring boards" OR "data monitoring board” OR "data safety monitoring boards" OR "data safety monitoring board" OR "data safety monitoring committee" OR "data safety monitoring committees" OR "data safety and monitoring boards" OR "data safety and monitoring board" OR "data and safety monitoring board" OR "independent data monitoring boards" OR "independent data monitoring board" OR "independent data monitoring committees" OR "independent data monitoring committee" OR "independent data safety monitoring committees" OR "independent data safety monitoring committee")) AND LANGUAGE: (English)  *Indexes=SCI-EXPANDED Timespan=All years* |
| #1 | 98 | (TI=("data monitoring committees" OR "data monitoring committee" OR "data monitoring boards" OR "data monitoring board” OR "data safety monitoring boards" OR "data safety monitoring board" OR "data safety monitoring committee" OR "data safety monitoring committees" OR "data safety and monitoring boards" OR "data safety and monitoring board" OR "data and safety monitoring board" OR "independent data monitoring boards" OR "independent data monitoring board" OR "independent data monitoring committees" OR "independent data monitoring committee" OR "independent data safety monitoring committees" OR "independent data safety monitoring committee")) *AND* LANGUAGE: (English) *Indexes=SCI-EXPANDED Timespan=All years* |

**Search Strategy for *EMBASE* (Decmeber 2015)**

The EMBASE database for all articles pertaining to discussion of Data Safety Monitoring Boards from inception of the database to December 2015, through OVID technologies.

1271 citations were found using the search strategy below.

The following was the search strategy used:

| **Search Number** | **Number of**  **Citations Found** | **Search Query** |
| --- | --- | --- |
| #2 | 1271 | Limit 1 to english language |
| #1 | 1290 | ("data monitoring committees" or "data monitoring committee" or "data safety monitoring boards" or "data safety monitoring board" or "data safety monitoring committee" or "data safety monitoring committees" or "data safety and monitoring boards" or "data safety and monitoring board" or "data and safety monitoring board" or "independent data monitoring boards" or "independent data monitoring board" or "independent data monitoring committees" or "independent data monitoring committee" or "independent data safety monitoring committees" or "independent data safety monitoring committee" or "data monitoring committees" or "data monitoring committee").ab. or ("data monitoring committees" or "data monitoring committee" or "data safety monitoring boards" or "data safety monitoring board" or "data safety monitoring committee" or "data safety monitoring committees" or "data safety and monitoring boards" or "data safety and monitoring board" or "data and safety monitoring board" or "independent data monitoring boards" or "independent data monitoring board" or "independent data monitoring committees" or "independent data monitoring committee" or "independent data safety monitoring committees" or "independent data safety monitoring committee" or "data monitoring committees" or "data monitoring committee").ti. or ("data monitoring committees" or "data monitoring committee" or "data safety monitoring boards" or "data safety monitoring board" or "data safety monitoring committee" or "data safety monitoring committees" or "data safety and monitoring boards" or "data safety and monitoring board" or "data and safety monitoring board" or "independent data monitoring boards" or "independent data monitoring board" or "independent data monitoring committees" or "independent data monitoring committee" or "independent data safety monitoring committees" or "independent data safety monitoring committee" or "data monitoring committees" or "data monitoring committee").kw. |

**Search Strategy for *CINAHL* (December 2015)**

The *CINAHL* database for all articles pertaining to discussion of Data Safety Monitoring Boards from inception of the database to December 2015, through the EBSCOhost Research Databases Interface, Search Screen - Advanced Search, Database – CINAHL

128 citations were found using the search strategy below.

The following was the search strategy used:

| **Search Number** | **Number of**  **Citations Found** | **Search Query** | **Limiters and Expanders** |
| --- | --- | --- | --- |
| #1 | 128 | TI ( ("data monitoring committees" OR "data monitoring committee" OR "data monitoring boards" OR "data monitoring board” OR "data safety monitoring boards" OR "data safety monitoring board" OR "data safety monitoring committee" OR "data safety monitoring committees" OR "data safety and monitoring boards" OR "data safety and monitoring board" OR "data and safety monitoring board" OR "independent data monitoring boards" OR "independent data monitoring board" OR "independent data monitoring committees" OR "independent data monitoring committee" OR "independent data safety monitoring committees" OR "independent data safety monitoring committee") ) OR AB ( ("data monitoring committees" OR "data monitoring committee" OR "data monitoring boards" OR "data monitoring board” OR "data safety monitoring boards" OR "data safety monitoring board" OR "data safety monitoring committee" OR "data safety monitoring committees" OR "data safety and monitoring boards" OR "data safety and monitoring board" OR "data and safety monitoring board" OR "independent data monitoring boards" OR "independent data monitoring board" OR "independent data monitoring committees" OR "independent data monitoring committee" OR "independent data safety monitoring committees" OR "independent data safety monitoring committee") ) OR SU ( ("data monitoring committees" OR "data monitoring committee" OR "data monitoring boards" OR "data monitoring board” OR "data safety monitoring boards" OR "data safety monitoring board" OR "data safety monitoring committee" OR "data safety monitoring committees" OR "data safety and monitoring boards" OR "data safety and monitoring board" OR "data and safety monitoring board" OR "independent data monitoring boards" OR "independent data monitoring board" OR "independent data monitoring committees" OR "independent data monitoring committee" OR "independent data safety monitoring committees" OR "independent data safety monitoring committee") ) | - Limiters - English Language - Expanders - Apply related words; Also search within the full text of the articles; Apply equivalent subjects - Search modes - Boolean/Phrase |

**Search Strategy for the major governmental and regulatory funding bodies and guideline groups search (December 2015)**

For each of major, governmental regulatory/health research/funding bodies, and international guideline groups for health research listed further below, if they had a search feature for their website, the following key terms were used one at a time to find relevant material or documents on DSMBs or clinical trials:

1. data monitoring committees
2. data monitoring committee
3. data monitoring boards
4. data monitoring board
5. data safety monitoring boards
6. data safety monitoring board
7. data safety monitoring committee
8. data safety monitoring committees
9. data safety and monitoring boards
10. data safety and monitoring board
11. data and safety monitoring board
12. independent data monitoring boards
13. independent data monitoring board
14. independent data monitoring committees
15. independent data monitoring committee
16. independent data safety monitoring committees
17. independent data safety monitoring committee
18. Clinical Trials Data Monitoring Committees
19. clinical trials
20. randomized controlled trials
21. randomised controlled trials

One search strategy was to search each website with key terms within a search box, if this feature was available for the website. If one of the organizations listed above had a search box feature for their website, 21 key terms related to DSMBs or clinical trial research were used one at a time to find relevant material or documents or grey literature on DSMBs and clinical trials. The first 5 pages of the search results generated from the search box (if the feature was available) were reviewed for each of the search terms used. The other search strategy used to find relevant literature or grey literature was to explore the organization’s websites by clicking on relevant main headings and subheading respectively, related to clinical/medical research or DSMBs on the home page or index page of the organization’s website, if an index page was available.

Documents and pages dedicated to clinical trial research or DSMBs as indicated from the webpage’s or document’s title and abstract (if an abstract was available) were included for full text review from each of the 14 organization’s websites mentioned below. Documents and pages from the full text review were included for full text information extraction if there was discussion within the full text of the document or webpage about sharing interim trial data by the DSMB with parties outside of DSMB. This search strategy resulted in 73 webpages or documents included for the full text review in total and 27 webpages or documents included for full text information extraction.

**The following major, governmental regulatory/health research/funding bodies, and international guideline groups for health research were searched directly for relevant literature or information from their respective websites:**

**1) For the US**: *National Institutes of Health (NIH)* [1]. The search of the NIH website resulted in finding literature on policies and guidance for Data and Safety Monitoring of Clinical Trials from associated research divisions within the NIH. These associated research divisions included the following 17 divisions [2]:

1. *National Institute of Neurological Disorders and Stroke (NINDS) [3]*
2. *National Heart, Lung, and Blood Institute (NHLBI) [4]*
3. *National Eye Institute (NEI) [5]*
4. *National Cancer Institute (NCI) [6]*
5. *National Institute of Allergy and Infectious Diseases (NHLBI) [7]*
6. *National Institute on Alcohol Abuse and Alcoholism (NIAAA) [8]*
7. *National Institute on Aging (NIA) [9]*
8. *National Institute of Nursing Research (NINR) [10]*
9. *National Institute of Mental Health (NIMH) [11]*
10. *National Institute of General Medical Sciences (NIGMS) [12]*
11. *National Institute of Environmental Health Sciences (NIEHS) [13]*
12. *National Institute of Diabetes and Digestive and Kidney Diseases (NIDDK)[14]*
13. *National Institute of Dental and Craniofacial Research (NIDCR) [15]*
14. *National Institute on Deafness and Other Communication Disorders (NIDCD) [16]*
15. *National Institute on Drug Abuse (NIDA) [17]*
16. *National Institute of Child Health and Human Development (NICHD)[18]*
17. *National Centre for Complementary and Integrative Health (NCCIH) [19, 20]*

And the *U.S. Food and Drug Administration (FDA)* [21];

**2) For Canada:** *Health Canada* [22]*, Canadian Institutes for Health Research (CIHR)* [23], and *Panel on Research Ethics* [24]*;*

**3) For the UK:** *UK Department of Health* [25]*, Medical Research Council (MRC)* [26]*, National Institute for Health Research (NIHR)* [27]*,* and the *National Health Service; Health Research Authority (NHS HRA)* [28]

**4) For the European Union:** *European Medicines Agency (EMA)* [29]

**5) For Australia:** *The* *National Health and Medical Research Council* [30] and *Therapeutic Goods Administration* [31] via the Department of Health

**6) International Groups:** *International Conference on Harmonisation of Technical Requirements for Registration of Pharmaceuticals for Human Use (ICH)* [32], which is a group that brings together regulatory authorities and the pharmaceutical industry to discuss the science and technical parts of drug registration and medical studies, and the *World Health Organization (WHO )* [33]

**References**

1. **National Institutes of Health**

[<http://www.nih.gov/>]

2. **NIH Policies and IC Guidance for Data and Safety Monitoring of Clinical Trials** [<http://grants.nih.gov/grants/policy/hs/data_safety.htm>]

3. **NINDS Guidelines for Data and Safety Monitoring in Clinical Trials** [<http://www.ninds.nih.gov/research/clinical_research/policies/data_safety_monitoring.htm>]

4. **NHLBI Policy for Data and Safety Monitoring of Extramural Clinical Studies** [<http://www.nhlbi.nih.gov/research/funding/human-subjects/data-safety-monitoring-policy>]

5. **National Eye Institute Guidelines for Data and Safety Monitoring of Clinical Trials** [<https://nei.nih.gov/funding/policy/policy6>]

6. **Essential Elements of a Data and Safety Monitoring Plan for Clinical Trials Funded by the National Cancer Institute** [<http://rrp.cancer.gov/clinicalTrials/data_safety_monitoring_plan.htm>]

7. **NHLBI Policy for Data and Safety Monitoring of Extramural Clinical Studies** [<http://www.niaid.nih.gov/labsandresources/resources/toolkit/guidance/Pages/guidance.aspx>]

8. **Data and Safety Monitoring Guidelines** [<http://www.niaaa.nih.gov/ResearchInformation/ExtramuralResearch/ResourcesAppGrantees/guidelines.htm>]

9. **Implementation of Policies for Human Intervention Studies** [<https://www.nia.nih.gov/research/dea/implementation-policies-human-intervention-studies>]

10. **Policy of the National Institute of Nursing Research for Data and Safety Monitoring of Extramural Clinical Trials** [<https://www.ninr.nih.gov/sites/www.ninr.nih.gov/files/NINR%20DSM%20Policy%202014%20FINAL.pdf>]

11. **NIMH Policy Governing the Monitoring of Clinical Trials** [<http://www.nimh.nih.gov/funding/clinical-research/nimh-policy-governing-the-monitoring-of-clinical-trials.shtml>]

12. **NIGMS Guidelines for Data and Safety Monitoring in Clinical Trials** [<http://www.nigms.nih.gov/Research/bioethics/Pages/clinicaltrials.aspx>]

13. **Policy for Data and Safety Monitoring of Human Subject Research Studies** [<http://www.niehs.nih.gov/research/clinical/patientprotections/dsmb/index.cfm>]

14. **Data & Safety Monitoring Plans** [<http://www.niddk.nih.gov/research-funding/process/human-subjects-research/policies-for-clinical-researchers/data-safety-monitoring-plans/Pages/data-and-safety-monitoring-plans.aspx>]

15. **NIDCR Policy for Data and Safety Monitoring of Clinical Research** [<http://www.nidcr.nih.gov/Research/ToolsforResearchers/Toolkit/DataandSafetyMonitoring.htm>]

16. **NIDCD Guidelines for Data and Safety Monitoring of Clinical Trials** [<http://www.nidcd.nih.gov/research/clinicalstudies/Information-for-Researchers-and-Health-Professionals/Pages/NIDCD-Guidelines-for-Data-and-Safety-Monitoring-of-Clinical-Trials.aspx>]

17. **Guidelines for Developing a Data and Safety Monitoring Plan** [<http://www.drugabuse.gov/funding/clinical-research/guidelines-developing-data-safety-monitoring-plan>]

18. **Data and Safety Monitoring Guidelines** [<http://www.niams.nih.gov/Funding/Clinical_Research/data_safety_monitoring_guidelines.pdf>]

19. **Data and Safety Monitoring of NCCIH-Funded Clinical Research** [<https://nccih.nih.gov/grants/policies/data-safety-monitoring>]

20. **Guidelines for NCCIH-Appointed Data and Safety Monitoring Boards** [<https://nccih.nih.gov/research/policies/datasafety>]

21. **U.S. Food and Drug Administration** [<http://www.fda.gov/>]

22. **Health Canada** [<http://www.hc-sc.gc.ca/index-eng.php>]

23. **Canadian Institutes of Health Research** [<http://cihr-irsc.gc.ca/e/193.html>]

24. **Panel on Research Ethics** [<http://www.pre.ethics.gc.ca/eng/index/>]

25. **UK Department of Health** [<https://www.gov.uk/government/organisations/department-of-health>]

26. **Medical Research Council** [<http://www.mrc.ac.uk/>]

27. **National Institutes for Health Research** [<http://www.nihr.ac.uk/>]

28. **National Health Service** [<http://www.nhs.uk/Pages/HomePage.aspx>]

29. **European Medicines Agency** [<http://www.ema.europa.eu/ema/>]

30. **National Health and Medical Research Council** [<http://www.nhmrc.gov.au/>]

31. **Therapeutic Goods Administration** [<http://www.tga.gov.au/>]

32. **International Conference on Harmonisation of Technical Requirements for Registration of Pharmaceuticals for Human Use** [<http://www.ich.org/home.html>]

33. **Who we are** [<http://www.who.int/about/who-we-are/en/>]
