# Supplementary material for: Sharing interim trial results by the Data Safety Monitoring Board with those responsible for the trial’s conduct and progress: a narrative review
Source: Trials. 2017 Mar 9;18:120. doi: 10.1186/s13063-017-1858-y (PMC5345177; doi:10.1186/s13063-017-1858-y)
Supplement: Additional file 2: — Citations of included articles for review. (DOCX 33 kb) [file 13063_2017_1858_MOESM2_ESM.docx]

**Additional file 2: Citations of included articles for review**

1. Whitehead J: **On being the statistician on a Data and Safety Monitoring Board**. In: *Stat Med. Volume 18*, edn. England: 1999 John Wiley & Sons, Ltd.; 1999: 3425-3434.

2. Sydes MR, Spiegelhalter DJ, Altman DG, Babiker AB, Parmar MK: **Systematic qualitative review of the literature on data monitoring committees for randomized controlled trials**. *Clin Trials* 2004, **1**(1):60-79.

3. DeMets DL, Fleming TR, Whitley RJ, Childress JF, Ellenberg SS, Foulkes M, Mayer KH, O'Fallon J, Pollard RB, Rahal JJ *et al*: **The data and safety monitoring board and acquired immune deficiency syndrome (AIDS) clinical trials**. In: *Control Clin Trials. Volume 16*, edn. United States; 1995: 408-421.

4. Fleming TR, Hennekens CH, Pfeffer MA, DeMets DL: **Enhancing trial integrity by protecting the independence of data monitoring committees in clinical trials**. *J Biopharm Stat* 2014, **24**(5):968-975.

5. Miller FG, Wendler D: **Is it ethical to keep interim findings of randomised controlled trials confidential?** In: *J Med Ethics. Volume 34*, edn. England; 2008: 198-201.

6. Sleight P: **Where are clinical trials going? Society and clinical trials**. *Journal of Internal Medicine* 2004, **255**(2):151-158.

7. Eckstein L: **Building a More Connected DSMB: Better Integrating Ethics Review and Safety Monitoring**. *Accountability in Research-Policies and Quality Assurance* 2015, **22**(2):81-105.

8. Ellenberg SS: **Monitoring data on data monitoring**. *Clin Trials* 2004, **1**(1):6-8.

9. Ellenberg SS, George SL: **Should statisticians reporting to data monitoring committees be independent of the trial sponsor and leadership?** *Stat Med* 2004, **23**(10):1503-1505.

10. Fleming TR: **Standard versus adaptive monitoring procedures: A commentary**. *Stat Med* 2006, **25**(19):3305-3312; discussion 3313-3304, 3326-3347.

11. Freidlin B, Korn EL: **Release of data from an ongoing randomized clinical trial for sample size adjustment or planning**. *Statistics in Medicine* 2007, **26**(22):4074-4082.

12. Glover JM, Kay R: **Who Advises the Data Monitoring Committee (DMC)? A Review of Regulatory Guidance for Sponsors on DMCs After 5 Years and Advice for DMC Members**. *Drug Information Journal* 2012, **46**(5):525-531.

13. London AJ: **Commentary: Data monitoring confidentiality and FDA transparency**. *Clinical Trials* 2014.

14. Richards SM: **Monitoring clinical trials. Interim data should not be publicly available**. *BMJ* 2001, **323**(7326):1424.

15. Schwartz PJ, Bigger JT, Breithardt G, Camm AJ, Demets DL, Furberg CD, Hallstrom A, Janse MJ, Julian DG, Lan KKG *et al*: **The early termination of clinical trials – causes, consequences, and control – with special references to trial in the field of arrhythmias and sudden death.** *Circulation* 1994, **89**(6):2892-2907.

16. Simon R: **Some practical aspects of the interim monitoring of clinical trials**. *Stat Med* 1994, **13**(13-14):1401-1409.

17. Slutsky AS, Lavery JV: **Data safety and monitoring boards**. *New England Journal of Medicine* 2004, **350**(11):1143-1147.

18. Slutsky AS, Lavery JV: **Data safety and monitoring boards - Reply**. *New England Journal of Medicine* 2004, **350**(23):2423-2423.

19. Thatte U, Kulkarni-Munshi R: **Data Safety Monitoring Boards**. *Natl Med J India* 2007, **20**(4):165-168.

20. Whitehead J: **Monitoring and evaluating clinical trials data**. *Intensive Care Med* 2000, **26 Suppl 1**:S84-88.

21. Abraham E: **Better infrastructure: industry-academia partnerships--a marriage of convenience?** *Crit Care Med* 2009, **37**(1 Suppl):S159-164.

22. Day S: **Data monitoring committees in clinical trials: Best practice, complexities and considerations**. *Clinical Investigation* 2015, **5**(7):615-617.

23. Ellenberg S: **The use of data monitoring committees in clinical trials**. *Drug Information Journal* 1996, **30**(2):553-557.

24. Friedman L, DeMets D: **The data monitoring committee: how it operates and why**. *Irb* 1981, **3**(4):6-8.

25. Thornton H: **Monitoring clinical trials. Caution may be warranted in releasing interim trial data**. *Bmj* 2001, **323**(7326):1424-1425.

26. Parry D: **Monitoring clinical trials. Several points are contentious**. *Bmj* 2001, **323**(7326):1425.

27. Gordon VM, Sugarman J, Kass N: **Toward a more comprehensive approach to protecting human subjects: the interface of data safety monitoring boards and institutional review boards in randomized clinical trials**. *IRB* 1998, **20**(1):1-5.

28. Tharmanathan P, Calvert MJ, Freemantle N: **Striding forward or getting too big for their boots? The developing role of data monitoring committees in clinical trials**. In: *J Clin Pharm Ther. Volume 31*, edn. England; 2006: 111-118.

29. Williams GW: **The other side of clinical trial monitoring; assuring data quality and procedural adherence**. In: *Clin Trials. Volume 3*, edn. England; 2006: 530-537.

30. Leon AC: **Independent data and safety monitoring in psychiatric intervention research**. *J Clin Psychiatry* 2012, **73**(2):e257-263.

31. Facey KM, Lewis JA: **The management of interim analyses in drug development**. In: *Stat Med. Volume 17*, edn. England; 1998: 1801-1809; discussion 1811-1802.

32. Ball G, Piller LB, Silverman MH: **Continuous safety monitoring for randomized controlled clinical trials with blinded treatment information Part 1: Ethical considerations.** *Contemporary Clinical Trials*  2011, **32**:S2-S4.

33. Novack GD: **Data Monitoring Committees**. *Ocul Surf* 2010, **8**(1):40-43.

34. Bloudoff-Indelicato M: **Threat of interim data leaks prompts call for international rules**. In: *Nat Med. Volume 21*, edn. United States; 2015: 200.

35. Chow SC, Corey R, Lin M: **On the independence of data monitoring committee in adaptive design clinical trials**. *J Biopharm Stat* 2012, **22**(4):853-867.

36. Anand SS, Wittes J, Yusuf S: **What information should a sponsor of a randomized trial receive during its conduct?** In: *Clin Trials. Volume 8*, edn. England; 2011: 716-719.

37. Grant AM, Altman DG, Babiker AG, Campbell MK, Clemens F, Darbyshire JH, Elbourne DR, McLeer SK, Parmar MKB, Pocock SJ *et al*: **A proposed charter for clinical trial data monitoring committees: helping them to do their job well**. *Lancet* 2005, **365**(9460):711-722.

38. Tereskerz PM, Guterbock TM, Kermer DA, Moreno JD: **An opinion and practice survey on the structure and management of data and safety monitoring boards**. In: *Account Res. Volume 18*, edn. United States; 2011: 1-30.

39. DeAngelis CD, Fontanarosa PB: **Ensuring integrity in industry-sponsored research: primum non nocere, revisited**. In: *JAMA. Volume 303*, edn. United States; 2010: 1196-1198.

40. Herson J: **Coordinating data monitoring committees and adaptive clinical trial designs**. *Drug Information Journal* 2008, **42**(4):297-301.

41. Smith MA, Ungerleider RS, Korn EL, Rubinstein L, Simon R: **Secrecy for Data Monitoring Committees: Inferior ethics, bad policy - Reply**. *Journal of Clinical Oncology* 1998, **16**(1):391-391.

42. Fisher MR, Roecker EB, DeMets DL: **The role of an independent statistical analysis center in the industry-modified National Institutes of Health model**. *Drug Information Journal* 2001, **35**(1):115-129.

43. Grant A: **Stopping clinical trials early**. In: *BMJ. Volume 329*, edn. England; 2004: 525-526.

44. Hilbrich L, Sleight P: **Progress and problems for randomized clinical trials: from streptomycin to the era of megatrials**. In: *Eur Heart J. Volume 27*, edn. England; 2006: 2158-2164.

45. Bergsjo P, Breart G, Morabia A: **Monitoring data and safety in the WHO Antenatal Care Trial**. *Paediatric and Perinatal Epidemiology* 1998, **12**:156-164.

46. Bath PM, Gray LJ, Wahlgren NG: **Should data monitoring committees assess efficacy when considering safety in trials in acute stroke?** In: *Int J Clin Pract. Volume 61*, edn. England; 2007: 1749-1755.

47. Brass EP: **Implementation of a data and safety monitoring program in a general clinical research center**. *J Investig Med* 2001, **49**(6):479-485.

48. Califf RM: **Watching the WATCH trial: the role of sponsors and data monitoring committees**. In: *J Card Fail. Volume 10*, edn. United States; 2004: 113-114.

49. D'Agostino RB, Sr., Massaro JM: **New developments in medical clinical trials**. In: *J Dent Res. Volume 83 Spec No C*, edn. United States; 2004: C18-24.

50. Ellenberg S, Crowley J, Meinert C, George S, Pocock S, Armitage P, Simon R, Yusuf S, Wittes J: **A survey of monitoring practice in cancer trial – data monitoring committees for Southwest Oncology Group clinicail trials – Discussion.** *Statistics in Medicine* 1993, **12**(5-6):457-459.

51. Korn EL, Freidlin B: **Inefficacy interim monitoring procedures in randomized clinical trials: the need to report**. In: *Am J Bioeth. Volume 11*, edn. England; 2011: 2-10.

52. Dixon DO: **Commentary on Anand et al**. In: *Clin Trials. Volume 8*, edn. England; 2011: 720-721; discussion 726.

53. Drazen JM, Wood AJ: **Don't mess with the DSMB**. In: *N Engl J Med. Volume 363*, edn. United States; 2010: 477-478.

54. Rockhold FW, Enas GG: **Data monitoring and interim analyses in the pharmaceutical industry: ethical and logistical considerations**. *Stat Med* 1993, **12**(5-6):471-479.

55. Hemmings R, Day S: **Regulatory perspectives on data safety monitoring boards: protecting the integrity of data**. In: *Drug Saf. Volume 27*, edn. New Zealand; 2004: 1-6.

56. Fleming TR, Sharples K, McCall J, Moore A, Rodgers A, Stewart R: **Maintaining confidentiality of interim data to enhance trial integrity and credibility**. In: *Clin Trials. Volume 5*, edn. England; 2008: 157-167.

57. George SL: **A survey of monitoring practices in cancer clinical trials**. *Stat Med* 1993, **12**(5-6):435-450.

58. Broglio KR, Stivers DN, Berry DA: **Predicting clinical trial results based on announcements of interim analyses**. In: *Trials. Volume 15*, edn. England; 2014: 73.

59. Silverman H: **Ethical issues during the conduct of clinical trials**. In: *Proc Am Thorac Soc. Volume 4*, edn. United States; 2007: 180-184; discussion 184.

60. Borer JS, Gordon DJ, Geller NL: **When should data and safety monitoring committees share interim results in cardiovascular trials?** In: *JAMA. Volume 299*, edn. United States; 2008: 1710-1712.

61. Califf RM: **Commentary on Anand et al**. In: *Clin Trials. Volume 8*, edn. England; 2011: 724-725; discussion 726.

62. DeMets DL: **Commentary on Anand et al**. In: *Clin Trials. Volume 8*, edn. England; 2011: 722-723; discussion 726.

63. Agency EM: **Committee for Medicinal Products for Human Use (CHMP) guideline on Data Monitoring Committees**. *Stat Med* 2006, **25**(10):1639-1645.

64. **Policy of the National Institute of Nursing Research for Data and Safety Monitoring of Extramural Clinical Trials** [https://www.ninr.nih.gov/sites/www.ninr.nih.gov/files/NINR%20DSM%20Policy%202014%20FINAL.pdf]

65. **Guidelines for NCCIH-Appointed Data and Safety Monitoring Boards** [https://nccih.nih.gov/research/policies/datasafety]

66. **Confidentiality of Interim Results in Cardiovascular (CV) Outcomes Safety Trials; Part 15 - PUBLIC HEARING BEFORE THE COMMISSIONER; Request for Comments** [http://www.fda.gov/Drugs/NewsEvents/ucm405023.htm]

67. **National Eye Institute Guidelines for Data and Safety Monitoring of Clinical Trials** [https://nei.nih.gov/funding/policy/policy6]

68. **NIDCD Guidelines for Data and Safety Monitoring of Clinical Trials** [http://www.nidcd.nih.gov/research/clinicalstudies/Information-for-Researchers-and-Health-Professionals/Pages/NIDCD-Guidelines-for-Data-and-Safety-Monitoring-of-Clinical-Trials.aspx]

69. Grant AM, Altman DG, Babiker AB, Campbell MK, Clemens FJ, Darbyshire JH, Elbourne DR, McLeer SK, Parmar MK, Pocock SJ *et al*: **Issues in data monitoring and interim analysis of trials**. In: *Health Technol Assess. Volume 9*, edn. England; 2005: 1-238, iii-iv.

70. Administration USFaD: **FDA Guidance on Clinical Trial Dat Monitoring Committee (DMCs)**. In*.* Rockville, MD: U.S Food and Drug Administration; 2001: 237.

71. Health NIo: **NIH Policy for Data and Safety Monitoring**. In*.* Edited by Health NIo. Bethesda, Maryland: National Institutes of Health; 1998.

72. Wells RJ: **Secrecy for data monitoring committees: inferior ethics, bad policy**. *J Clin Oncol* 1998, **16**(1):390-391.

73. Lilford RJ, Braunholtz D, Edwards S, Stevens A: **Monitoring clinical trials - interim data should be publicly available**. *British Medical Journal* 2001, **323**(7310):441-442.

74. Morse MA, Califf RM, Sugarman J: **Monitoring and ensuring safety during clinical research**. In: *JAMA. Volume 285*, edn. United States; 2001: 1201-1205.

75. Vail A, Hornbuckle J, Spiegelhalter DJ, Thornton JG: **Prospective application of Bayesian monitoring and analysis in an 'open' randomized clinical trial**. *Statistics in Medicine* 2001, **20**(24):3777-3787.

76. Wells RJ, Gartside PS, McHenry CL: **Ethical issues arising when interim data in clinical trials is restricted to independent data monitoring committees**. *IRB* 2000, **22**(1):7-11.

77. Hanna KE: **The other oversight committees: Data Monitoring Committees and Institutional Biosafety Committees**. *Research Practitioner* 2004, **5**(3):83-93 11p.

78. Henk JM: **Monitoring clinical trials. Latest data from START trial should be made available**. *Bmj* 2001, **323**(7326):1425-1426.

79. Geller NL, Stylianou M: **Practical issues in data monitoring of clinical trials: summary of responses to a questionnaire at NIH**. *Stat Med* 1993, **12**(5-6):543-551; discussion 553.

80. Wells RJ: **Data safety and monitoring boards**. In: *N Engl J Med. Volume 350*, edn. United States; 2004: 2423;; author reply 2423.

81. Bryant J: **What is the appropriate role of the trial statistician in preparing and presenting interim findings to an independent Data Monitoring Committee in the U.S. Cancer Cooperative Group setting?** *Stat Med* 2004, **23**(10):1507-1511.

82. Armitage P, Delta Data Safety Monitoring C: **Data and safety monitoring in the Delta trial**. *Controlled Clinical Trials* 1999, **20**(3):229-241.

83. Califf RM, Ellenberg SS: **Statistical approaches and policies for the operations of Data and Safety Monitoring Committees**. *American Heart Journal* 2001, **141**(2):301-305.

84. Cuzick J, Howell A, Forbes J: **Early stopping of clinical trials**. *Breast Cancer Research* 2005, **7**(5):181-183.

85. Chalmers I, Altman DG, McHaffie H, Owens N, Cooke RW: **Data sharing among data monitoring committees and responsibilities to patients and science**. In: *Trials. Volume 14*, edn. England; 2013: 102.

86. Shah SK, Dawson L, Dixon DO, Lie RK: **Should sponsors and DSMBs share interim results across trials?** *J Acquir Immune Defic Syndr* 2011, **58**(5):433-435.

87. Health NIo: **Further guidance on a data and safety monitoring for phase I and phase II trials** In*.* Edited by Health NIo. Bethesda, Maryland: National Institutes of Health; 2000.

88. **NIGMS Guidelines for Data and Safety Monitoring in Clinical Trials** [http://www.nigms.nih.gov/Research/bioethics/Pages/clinicaltrials.aspx]

89. **Data and Safety Monitoring Guidelines** [http://www.niaaa.nih.gov/ResearchInformation/ExtramuralResearch/ResourcesAppGrantees/guidelines.htm]

90. Coutant D: **Interim Analysis: What Regulatory Affairs Professionals Need to Know**. *Drug Information Journal* 2010, **44**(6):703-712.

91. Herson J: **Data and safety monitoring committees in clinical trials**. Boca Raton: Chapman & Hall/CRC/Taylor & Francis; 2009.

92. Ellenberg SS, Fleming TR, DeMets DL: **Data monitoring committees in clinical trials: a practical perspective**: John Wiley & Sons; 2003.

93. Green S, Crowley J: **Data Monitoring Committees for Southwest Oncology Group clinical trials.** *Statistics in Medicine* 1993, **12**(5-6):451-455.

94. Nissen SE: **Commentary: confidentiality of interim trial data-the emerging crisis**. *Clin Trials* 2015, **12**(1):15-17.

95. Packer M, Wittes J, Stump D: **Terms of reference for Data and Safety Monitoring Committees**. *American Heart Journal* 2001, **141**(4):542-547.

96. Pocock S, Furberg CD: **Procedures of Data and Safety Monitoring Committees**. *American Heart Journal* 2001, **141**(2):289-294.

97. Pocock SJ: **A major trial needs three statisticians: why, how and who?** *Stat Med* 2004, **23**(10):1535-1539.

98. Rouse DJ: **Data monitoring and safety committees and their operations**. *Obstet Gynecol Surv* 2003, **58**(5):329-336.

99. Sartor O, Halabi S: **Independent data monitoring committees: An update and overview**. *Urol Oncol* 2015, **33**(3):143-148.

100. Siegel JP, O'Neill RT, Temple R, Campbell G, Foulkes MA: **Independence of the statistician who analyses unblinded data**. *Stat Med* 2004, **23**(10):1527-1529.

101. Smith MA, Ungerleider RS, Korn EL, Rubinstein L, Simon R: **Role of independent data-monitoring committees in randomized clinical trials sponsored by the National Cancer Institute**. *J Clin Oncol* 1997, **15**(7):2736-2743.

102. Wilhelmsen L: **Role of the Data and Safety Monitoring Committee (DSMC)**. *Stat Med* 2002, **21**(19):2823-2829.

103. Williams GW, Davis RL, Getson AJ, Gould AL, Hwang IK, Matthews H, Shih WCJ, Snapinn SM, Waltonbowen KL: **Monitoring of clinical trials and interim analyses from a drug sponsors point-of-view.** *Statistics in Medicine* 1993, **12**(5-6):481-492.

104. Wittes J: **Forming your phase III trial's data and safety monitoring board: a perspective on safety**. *J Investig Med* 2004, **52**(7):453-458.

105. Wittes J, Schactman M: **On independent data monitoring committees in oncology clinical trials**. *Chin Clin Oncol* 2014, **3**(3):40.

106. Gallo P: **Operational challenges in adaptive design implementation**. *Pharmaceutical Statistics* 2006, **5**(2):119-124.

107. Gallo P: **Confidentiality and trial integrity issues for adaptive designs**. *Drug Information Journal* 2006, **40**(4):445-450.

108. Ellenberg SS, Myers MW, Blackwelder WC, Hoth DF: **The use of external monitoring committees in clinical trial of the National Institute of Allergy and Infectious Diseases.** *Statistics in Medicine* 1993, **12**(5-6):461-467.

109. Fleming TR, Ellenberg S, DeMets DL: **Monitoring clinical trials: issues and controversies regarding confidentiality**. *Stat Med* 2002, **21**(19):2843-2851.

110. Fleming TR: **Protecting the confidentiality of interim data: Addressing current challenges**. *Clinical Trials* 2015, **12**(1):5-11.

111. Walters L: **Data monitoring committees: the moral case for maximum feasible independence**. *Stat Med* 1993, **12**(5-6):575-580.

112. Yusuf, Whitley R, Assenzo R, Fleming T, Deykin, Hawkins B, Meier, Gent, Tognoni, Ellenberg J *et al*: **The operation of data monitoring committees – discussion.** *Statistics in Medicine* 1993, **12**(5-6):527-542.

113. Ellenberg SS: **Protecting clinical trial participants and protecting data integrity: are we meeting the challenges?** In: *PLoS Med. Volume 9*, edn. United States; 2012: e1001234.

114. Chen-Mok M, VanRaden MJ, Higgs ES, Dominik R: **Experiences and challenges in data monitoring for clinical trials within an international tropical disease research network**. In: *Clin Trials. Volume 3*, edn. England; 2006: 469-477.

115. Fleming TR, DeMets DL: **Monitoring of clinical trials: issues and recommendations**. In: *Control Clin Trials. Volume 14*, edn. United States; 1993: 183-197.

116. D'Agostino RB, Sr.: **The statistician and the data monitoring committee**. *Stat Med* 2004, **23**(10):1501-1502.

117. Allardyce RA, Bagshaw PF, Frampton CM, Frizelle FA, Hewett PJ, Rieger NA, Smith JS, Solomon MJ, Stevenson AR: **Ethical issues with the disclosure of surgical trial short-term data**. *ANZ J Surg* 2011, **81**(3):125-131.

118. Antonijevic Z, Gallo P, Chuang-Stein C, Dragalin V, Loewy J, Menon S, Miller ER, Morgan CC, Sanchez M: **Views on Emerging Issues Pertaining to Data Monitoring Committees for Adaptive Trials**. *Therapeutic Innovation & Regulatory Science* 2013, **47**(4):495-502.

119. Kowalski CJ, Hewett JL: **Data and safety monitoring boards: some enduring questions**. In: *J Law Med Ethics. Volume 37*, edn. United States; 2009: 496-506, 396-497.

120. Baggs GE, Seth A, Oliver JS, Jones WM, Liu L, Toth SM: **Monitoring clinical trial data using an unblinded industry statistician**. *Drug Information Journal* 2008, **42**(2):193-199.

121. DeMets DL: **Relationships between Data Monitoring Committees**. *Controlled Clinical Trials* 2000, **21**(1):54-55.

122. DeMets D, Califf R, Dixon D, Ellenberg S, Fleming T, Held P, Julian D, Kaplan R, Levine R, Neaton J *et al*: **Issues in regulatory guidelines for data monitoring committees**. *Clin Trials* 2004, **1**(2):162-169.

123. Ellenberg SS: **Independent data monitoring committees: rationale, operations and controversies**. In: *Stat Med. Volume 20*, edn. England; 2001: 2573-2583.

124. Gallo P, Chuang-Stein C, Dragalin V, Gaydos B, Krams M, Pinheiro J: **Adaptive designs in clinical drug development--an Executive Summary of the PhRMA Working Group**. *J Biopharm Stat* 2006, **16**(3):275-283; discussion 285-291, 293-278, 311-272.

125. Migrino RQ, Topol EJ: **A matter of life and death? The Heart Protection Study and protection of clinical trial participants**. *Controlled Clinical Trials* 2003, **24**(5):501-505.

126. Gallo P, Fardipour P, Dragalin V, Krams M, Littman GS, Bretz F: **Data Monitoring in Adaptive Dose-Ranging Trials**. *Statistics in Biopharmaceutical Research* 2010, **2**(4):513-521.

127. Taylor HA, Chaisson L, Sugarman J: **Enhancing communication among data monitoring committees and institutional review boards**. In: *Clin Trials. Volume 5*, edn. England; 2008: 277-282.

128. Wittes J, Barrett-Connor E, Braunwald E, Chesney M, Cohen HJ, Demets D, Dunn L, Dwyer J, Heaney RP, Vogel V *et al*: **Monitoring the randomized trials of the Women's Health Initiative: the experience of the Data and Safety Monitoring Board**. In: *Clin Trials. Volume 4*, edn. England; 2007: 218-234.

129. Korn EL, Hunsberger S, Freidlin B, Smith MA, Abrams JS: **Comments on 'maintaining confidentiality of interim data to enhance trial integrity and credibility' by TR Fleming et al**. *Clin Trials* 2008, **5**(4):364-365; author reply 365-366.

130. Peppercorn J, Buss WG, Fost N, Godley PA: **The dilemma of data-safety monitoring: provision of significant new data to research participants**. *Lancet* 2008, **371**(9611):527-529.

131. Wittes J: **Playing safe and preserving integrity: making the FDA model work**. *Stat Med* 2004, **23**(10):1523-1525.

132. Lin JY, Lu Y: **Establishing a data monitoring committee for clinical trials**. *Shanghai archives of psychiatry* 2014, **26**(1):54-56.

133. Delgado-Herrera L, Anbar D: **A model for the interim analysis process: a case study**. In: *Control Clin Trials. Volume 24*, edn. United States; 2003: 51-65.

134. Cairns JA, Hallstrom A, Held P: **Should all trials have a Data Safety and Monitoring Committee?** *American Heart Journal* 2001, **141**(1):156-163.

135. Fardipour P, Littman G, Burns DD, Dragalin V, Padmanabhan SK, Parke T, Perevozskaya I, Reinold K, Sharma A, Krams M: **Planning and Executing Response-Adaptive Learn-Phase Clinical Trials: 1. The Process**. *Drug Information Journal* 2009, **43**(6):713-723.

136. Dixon DO, Lagakos SW: **Should data and safety monitoring boards share confidential interim data?** In: *Control Clin Trials. Volume 21*, edn. United States; 2000: 1-6; discussion 54-55.

137. Korn EL, Hunsberger S, Freidlin B, Smith MA, Abrams JS: **Preliminary data release for randomized clinical trials of noninferiority: a new proposal**. In: *J Clin Oncol. Volume 23*, edn. United States; 2005: 5831-5836.

138. Snapinn S, Cook T, Shapiro D, Snavely D: **The role of the unblinded sponsor statistician**. *Stat Med* 2004, **23**(10):1531-1533.

139. Armitage P: **Interim analysis in clinical trials**. *Stat Med* 1991, **10**(6):925-935; discussion 936-927.

140. Ginsberg MD, Palesch YY, Martin RH, Hill MD, Moy CS, Waldman BD, Yeatts SD, Tamariz D, Ryckborst K, Investigators A: **The Albumin in Acute Stroke (ALIAS) Multicenter Clinical Trial Safety Analysis of Part 1 and Rationale and Design of Part 2**. *Stroke* 2011, **42**(1):119-127.

141. Stephens RJ, Langley RE, Mulvenna P, Nankivell M, Vail A, Parmar MK: **Interim results in clinical trials: do we need to keep all interim randomised clinical trial results confidential?** *Lung cancer (Amsterdam, Netherlands)* 2014, **85**(2):116-118.

142. DeMets DL, Fleming TR: **The independent statistician for data monitoring committees**. *Stat Med* 2004, **23**(10):1513-1517.

143. Dixon DO, Weiss S, Cahill K, Fox L, Love J, McNamara J, Soto-Torres LE: **Data and safety monitoring policy for National Institute of Allergy and Infectious Diseases clinical trials**. In: *Clin Trials. Volume 8*, edn. England; 2011: 727-735.

144. Czaja SJ, Schulz R, Belle SH, Burgio LD, Armstrong N, Gitlin LN, Coon DW, Martindale-Adams J, Klinger J, Stahl SM: **Data and safety monitoring in social behavioral intervention trials: the REACH II experience**. *Clin Trials* 2006, **3**(2):107-118.

145. DeMets DL, Califf RM: **Lessons learned from recent cardiovascular clinical trials: Part II**. *Circulation* 2002, **106**(7):880-886.

146. Hillman DW, Louis TA: **DSMB case study: decision making when a similar clinical trial is stopped early**. In: *Control Clin Trials. Volume 24*, edn. United States; 2003: 85-91.

147. Hicks LK, Laupacis A, Slutsky AS: **A primer on data safety monitoring boards: mission, methods, and controversies**. *Intensive Care Med* 2007, **33**(10):1815-1818.

148. Administration USFaD: **Transcript for hearing: Part 15 Public Hearing; Confidentiality of Interim** In*.*, vol. 2015: U.S Food and Drug Administration; 2014.

149. Australian Government DoHTGA: **Note for guidance on clinical safety data management: definitions and standards for expedited reporting**. In*.* Edited by Adminstration TG, vol. 2015. Australia: Therapeutic Goods Administration; 2000: 13.

150. U.S Food and Drug Administration; Department of Health and Human Services; Park MR, C.; Jenkins, J.; Woodstock, J.: **Memorandum; Disclosure of Interim Cardiovascular Risk Study Data NDA 22271 Nesina (alogliptin) Tablets and its Fixed Dose Combination Product NDAs 22426 and 203414** In*.*, vol. 2013: U.S Food and Drug Administration; 2013.

151. International Council for Harmonisation of Technical Requirements for Pharmaceuticals for Human Use: **ICH Harmonised Tripartite Guideline; Statistical Prinicples for Clinical E9.** In*.*: International Council for Harmonisation of Technical Requirements for Pharmaceuticals for Human Use (ICH); 1998: 39.

152. US National Institutes of Health: **Guidance on reporting adverse events to institutional review boards for NIH-Supported multicentre clinical trials**. In*.* Edited by US National Institutes of Health. Bethesda, Maryland: National Institutes of Health; 1999.

153. US Food and Drug Adminstration: **Presentations for: Part 15 Public Hearing; Confidentiality of Interim Results in Cardiovascular (CV) Outcomes Safety Trials**. In*.* vol. 2015: U.S Food and Drug Administration; 2014.

154. World Health Organization: **Operational Guidelines for the Establishment and Functioning of Data and Safety Monitoring Boards**. In*.* Switzerland: World Health Organization 2005: 44.

155. **Data & Safety Monitoring Plans** [http://www.niddk.nih.gov/research-funding/process/human-subjects-research/policies-for-clinical-researchers/data-safety-monitoring-plans/Pages/data-and-safety-monitoring-plans.aspx]

156. **NINDS Guidelines for Data and Safety Monitoring in Clinical Trials** [http://www.ninds.nih.gov/research/clinical_research/policies/data_safety_monitoring.htm]

157. **NHLBI Policy for Data and Safety Monitoring of Extramural Clinical Studies** [http://www.niaid.nih.gov/labsandresources/resources/toolkit/guidance/Pages/guidance.aspx]

158. **Data and Safety Monitoring Guidelines** [http://www.niams.nih.gov/Funding/Clinical_Research/data_safety_monitoring_guidelines.pdf]

159. **NHLBI Policy for Data and Safety Monitoring of Extramural Clinical Studies** [http://www.nhlbi.nih.gov/research/funding/human-subjects/data-safety-monitoring-policy]

160. US Food and Drug Adminstration: **The Establishment and Operation of Clinical Trial Data Monitoring Committees for Clinical Trial Sponsors**. In*.*, vol. 2015. Rockville, MD: U.S Food and Drug Administration; 2006: 38.

161. **Implementation of Policies for Human Intervention Studies** [https://www.nia.nih.gov/research/dea/implementation-policies-human-intervention-studies]

162. **Policy for Data and Safety Monitoring of Human Subject Research Studies** [http://www.niehs.nih.gov/research/clinical/patientprotections/dsmb/index.cfm]
